# Public Support in the U.S. for Human‐Animal Chimera Research: Results of a Representative Cross‐Sectional Survey of 1,058 Adults

**DOI:** 10.1002/sctm.16-0452

**Published:** 2017-04-21

**Authors:** Jonathan Kantor

**Affiliations:** ^1^ Department of Dermatology, Perelman School of MedicineUniversity of Pennsylvania & Florida Center for Dermatology, P.A.Saint AugustineFloridaUSA

On August 4, 2016, the National Institutes of Health issued a request for public comment regarding relaxing recent restrictions on human‐animal stem cell research, which disallowed funding for any research involving the introduction of human pluripotent cells into animal embryos prior to gastrulation 1. The original restrictions took place in the context of a National Academy of Sciences recommendation that reflected concerns that implanted human pluripotent stem cells could contribute to the central nervous system or germline of target animals 2, 3.

The degree of public support for human‐animal stem cell research in the U.S. is unknown, despite the NIH controlling a $31 billion budget and the profound impact of NIH funding decisions on basic science research.

One recent study evaluating public support for chimera research in Japan found 49% of the Japanese public opposed to this research 2, and a similar degree of opposition has been cited in the U.K. as well 4.

In order to gauge the degree of public support for human‐animal chimera research in the U.S., a survey on these attitudes was developed after pilot testing using an iterative process (Supporting Information). The electronic survey was sent by Survey Monkey, a professional survey company, using a similar methodology to a recent report assessing popular attitudes to oocyte cryopreservation 5. Respondents were rewarded with a donation to a charity of their choice and a chance to win a gift card. Baseline responses were recorded and univariate unadjusted logistic regression odds ratios of association were assessed between respondent characteristics and a negative attitude to chimera research.

This study was deemed exempt from review by the local Institutional Review Board. All analyses were performed with Stata for Mac version 13 (College Station, TX).

Overall, 1,013 of 1,058 surveys were returned completed (95.7% completed response rate), and 22.6% of respondents were opposed to this research. Baseline demographic characteristics, degree of support for human‐animal chimera research, and association between these characteristics and a negative attitude to chimera research are listed in Table 1. Survey respondents constituted a representative geographic cross‐section of U.S. residents.

**Table 1:** Characteristics of respondents by attitude to human‐animal chimera research

Characteristic	In favor *N* = 554 (52.4%)	Neutral *N* = 265 (25.1%)	Opposed *N* = 239 (22.6%)	Odds ratio (95% confidence intervals)
Age—year	46.2 ± 17.4	48.8 ± 17.2	48.1 ± 15.4	NS
Male sex—no. (%)	306 (55.5)	109 (42.8)	68 (33.2)	0.47 (0.34, 0.65)
Race				NS
Black	21 (3.9)	14 (5.5)	8 (3.9)	
Asian	22 (4.0)	4 (1.6)	6 (2.9)	
Hispanic/Latino	24 (4.4)	13 (5.1)	9 (4.4)	
White	436 (80.0)	197 (77.9)	162 (79.4)	
Other/Unanswered	51 (9.2)	37 (14.0)	54 (22.6)	
Yearly income				NS
<$25,000	106 (19.2)	39 (15.3)	35 (17.1)	
$25,000–$49,999	98 (17.8)	53 (20.8)	50 (24.4)	
$50,000–$74,999	77 (14.0)	43 (16.9)	19 (9.3)	
>$75,000	214 (38.8)	75 (29.4)	72 (35.1)	
Unanswered	56 (10.2)	45 (17.7)	29 (14.2)	
Religion				NS
Atheist/Agnostic	176 (31.8)	47 (17.7)	48 (20.1)	
Catholic	79 (14.3)	49 (18.5)	31 (13.0)	
Jewish/Muslim/Hindu/Buddhist	39 (7.0)	22 (8.3)	11 (4.6)	
Protestant	187 (33.8)	100 (37.7)	81 (33.9)	
Other	73 (13.2)	47 (17.7)	68 (28.5)	
I consider myself a religious person				1.44 (1.08, 1.92)
Yes	256 (46.2)	143 (54.0)	138 (57.7)	
No	234 (42.2)	79 (29.8)	79 (33.1)	
Undecided	64 (11.6)	43 (16.2)	22 (9.2)	
Marital status				NS
Never Married	166 (30.3)	76 (29.7)	52 (25.7)	
Married	283 (51.6)	147 (57.4)	113 (55.9)	
Separated	11 (2.0)	2 (0.8)	4 (2.0)	
Divorced	64 (11.7)	23 (9.0)	23 (11.4)	
Widowed	24 (4.4)	8 (3.1)	10 (5.0)	
Education				NS
High School or Below	67 (12.1)	32 (12.1)	22(9.2)	
Some College	170 (30.7)	102 (38.5)	79 (33.1)	
Bachelor's	194 (35.0)	77 (29.1)	57 (23.9)	
Graduate Degree	120 (21.7)	45 (17.0)	48 (20.1)	
Unanswered	3 (0.5)	9 (3.4)	33 (13.8)	
Children at home				NS
Yes	215 (38.8)	92 (34.7)	74 (31.0)	
No	273 (49.3)	124 (46.8)	90 (37.7)	
Unanswered	66 (11.9)	49 (18.5)	75 (31.4)	
Pets at home				NS
Yes	359 (64.8)	160 (60.4)	163 (68.3)	
No	137 (24.7)	72 (27.2)	27 (11.3)	
Unanswered	58 (10.5)	33(12.5)	49 (20.5)	
Vegetarian				2.44 (1.54, 3.86)
Yes	38 (6.9)	20 (7.7)	33 (15.8)	
No	516 (93.1)	239 (92.3)	176 (84.2)	
Opposed to all animal research				9.92 (6.97, 14.12)
Yes	29 (5.2)	41 (15.5)	115 (48.1)	
No	427 (77.1)	110 (41.5)	98 (41.0)	
Undecided	98 (17.7)	114 (43.0)	26 (10.9)	
Organ donor				0.66 (0.50, 0.89)
Yes	370 (66.8)	159 (60.0)	131 (54.8)	
No	184 (33.2)	106 (40.0)	108 (45.2)	
Opinion regarding the number of daily deaths from organ shortage				NS
0	11 (2.0)	4 (1.5)	6 (2.6)	
<10	27 (4.9)	12 (4.5)	8 (3.4)	
11–99	110 (19.9)	45 (17.0)	43 (18.4)	
100–999	202 (36.5)	87 (32.8)	81 (34.6)	
>1,000	204 (36.8)	117 (44.2)	96 (41.0)	
Know someone with history of cancer				NS
Yes	444 (89.3)	201 (88.2)	173 (90.1)	
No	53 (10.7)	27 (11.8)	19 (9.9)	
Know someone with history of transplant				NS
Yes	159 (41.0)	80 (43.5)	61 (39.6)	
No	229 (59.0)	104 (56.5)	93 (60.4)	
I have thought about chimera research before				0.52 (0.37, 0.72)
Yes	245 (44.2)	42 (15.9)	52 (21.8)	
No	174 (31.4)	107 (40.4)	122 (51.1)	
Uncertain	135 (24.4)	116 (43.8)	65 (27.2)	
Central nervous system chimera research is appropriate				0.03 (0.01, 0.06)
Yes	429 (77.4)	19 (7.2)	8 (3.4)	
No	39 (7.0)	64 (24.2)	209 (87.5)	
Uncertain	86 (15.5)	182 (68.7)	22 (9.2)	

Data represent the number and percentage of subjects in favor, neutral, and opposed to chimera research within each subgroup. Odds ratios of association of various questionnaire responses with a negative attitude to chimera research.

Abbreviation: NS, not significant

These findings suggest that there is considerable support in the U.S. for human‐animal chimera research, and, importantly, that opposition to this research in the U.S. is markedly lower than seen in Japan and the U.K. While the surveys used in studies in the U.S., U.K., and Japan were not identical, all probed the general public's support for human‐animal chimera research.

As with all survey research, and online survey research in particular, there are important limitations to these preliminary findings, including generalizability, response bias, and social desirability bias. Online surveys of any sort may not fully generalize to the overall U.S. population. Mentioning organ transplantation in the survey introduction may have served to nudge respondents in a favorable direction, though this approach has been used by other similar surveys as well 1, 2, 3, 4 The high response rate seen in this study may be a function of the timeliness of the subject matter, as well as the methodology used by the professional survey company, though these results should be interpreted as preliminary in nature.

Since having previously contemplated chimera research was associated with an approximately 52% reduction in resistance to this research, outreach and education efforts may potentially have a further positive impact on mitigating public hesitancy regarding this field of research, though this remains to be definitively elucidated.

**Jonathan Kantor**
Department of Dermatology, Perelman School of Medicine, University of Pennsylvania & Florida Center for Dermatology,
P.A., Saint Augustine, Florida, USA

## Supporting information

Supporting Information
