# Supplementary material for: Public Support in the U.S. for Human‐Animal Chimera Research: Results of a Representative Cross‐Sectional Survey of 1,058 Adults
Source: Stem Cells Transl Med. 2017 Apr 21;6(5):1442–4. doi: 10.1002/sctm.16-0452 (PMC5442706; doi:10.1002/sctm.16-0452)
Supplement: Supplementary file 1 — Supporting Information [file SCT3-6-1442-s001.docx]

Very Short Human-Animal Stem Cell Questionnaire

There is a shortage of organs available for organ transplants.

To address this, research is planned that would implant human stem cells in the fertilized eggs of animals such as cows or rabbits to study human diseases and possibly create animals that possess human organs that can be used for transplantation.

Stem cells are able to turn into many different types of tissue, like heart, kidney, or nerve cells.

Research involving implanting human stem cells into other animals is sometimes called chimera research.

Remember, all of your answers are completely anonymous and there are no wrong answers!

This survey should take you less than 5 minutes.

Thanks for participating!

1. I am an organ donor

Yes

No

2. I think the number of people who die every day in the US while waiting for an organ transplant is:

None

Less than 10

Between 11 and 99

More than 100

More than 1,000

3. Someone I know has a history of cancer or organ transplant: (check all that apply):

I or someone I know has a history of cancer

I don’t know anyone with a history of cancer

I or someone I know has a history of organ transplant

I don’t know anyone with a history of organ transplant

4. I have thought about chimera research before?

Strongly agree

Agree

Undecided

Disagree

Strongly disagree

5. I think this research should be allowed on animals, as long as scientists agree that it is ethical and appropriate:

Strongly agree

Agree

Undecided

Disagree

Strongly disagree

6. I think the research should not be allowed on the following animals, even if scientists agree that it is ethically appropriate (check all that apply):

Laboratory animals like mice and rats

Domesticated animals like dogs and cats

Farm animals like pigs and cows

Primates like monkeys or chimpanzees

It should not be allowed on any animals

Other (please specify)

7. I think this research should be allowed when putting human stem cells into animal brains, as long as scientists agree that it is ethical and appropriate:

Strongly agree

Agree

Undecided

Disagree

Strongly disagree

8. I am vegetarian

Yes

No

9. I am opposed to any animal research of any kind

Strongly agree

Agree

Undecided

Disagree

Strongly disagree

1. I have/don’t have animals or pets living with me at home, and my age is:

I have no children

I have children

I have no pets

I have pets

My age is:

1. I would best describe my religion as:

Christian/ Protestant

Catholic

Jewish

Muslim

Hindu

Buddhist

Atheist

Agnostic

Other:

1. I consider myself a religious person:

Strongly agree

Agree

Undecided

Disagree

Strongly disagree

1. I would best describe my race as:

American Indian or Alaska Native (including all original peoples of the Americas)

Asian (including Indian subcontinent and Philippines)

Black or African American

Middle Eastern

Native Hawaiian or Pacific Islander

White or Caucasian

Hispanic or Latino

I would prefer not to answer this question

Other:

1. My highest level of education completed is:

Grade school

High school

Some college

Technical or associates degree

Bachelor’s degree (College degree)

Graduate or professional degree (MD, Ph.D., J.D.)

1. My marital status is

Never married

Married

Separated

Divorced

Widowed
